# Factors related to low birth rate among married women in Korea

**DOI:** 10.1371/journal.pone.0194597

**Published:** 2018-03-20

**Authors:** Ju-Eun Song, Jeong-Ah Ahn, Sun-Kyoung Lee, Eun Ha Roh

**Affiliations:** 1 Ajou University College of Nursing and Research Institute of Nursing Science, Suwon, South Korea; 2 Seoul Women’s College of Nursing, Seoul, South Korea; 3 Research Institute of Nursing Science, Ajou University, Suwon, South Korea; Western Sydney University, AUSTRALIA

## Abstract

The purpose of this study was to explore the factors influencing low birth rate among married women using the National Survey data in Korea. We compared the different influences on women’s first and subsequent childbirths. This study was a secondary analysis using the “National Survey on Fertility and Family Health and Welfare”, which was a nationally representative survey conducted by the Korea Institute for Health and Social Affairs. We analyzed the data of 3,482 married women (aged between 19 and 39 years) using SPSS 20.0 program for descriptive statistics, t-test, one-way ANOVA, and binary and ordinal logistic regression models. The factors influencing women’s first childbirth included perceptions about the value of marriage and children and their education level. The factors influencing their subsequent childbirths included multifaceted variables of maternal age during the first childbirth, residential area, religion, monthly household income, perceptions about the value of marriage and children, and social media. It is necessary to improve women’s awareness and positive perceptions about marriage and children in order to increase the birth rate in Korea. Moreover, consistently providing financial and political support for maternal and childcare concerns and using social media to foster more positive attitudes toward having children may enhance birth rates in the future.

## Introduction

Birth rates have globally fallen far below the replacement fertility rate of 2.1, especially in most developed and developing countries, and it is considered to be a critical issue regarding political challenges, economic growth, cultural stability, and more [1,2]. A generation of children turns into a generation of workers and parents. Declining fertility rate and further rapid contraction of the labor force may have negative effects on economic growth and on the ability of generations to support the society, such as paying taxes for facilitating pensions, education, health care services, and so on [3,4].

Recently, the total fertility or birth rate, which is the number of babies per woman over her lifetime, was recorded as 1.9 in the United States [5] and 1.2 in South Korea [6]. Korea has been experiencing a dramatic decline in the birth rate since 1960, when six children were born per woman, and now it is known as one of the countries with the lowest birth rate in the world [7]. Consequently, this low birth rate in Korea has further slowed the population growth and more rapidly accelerated the demographic transition to an aged society than any other OECD country [8]. As the number of children and young people decreases and the proportion of elderly people increases, continued decline in labor force participation rate will impact the country’s economic vitality including elevated cost of social and health care services [9].

An assumption underlying this drastic fertility transition is that economic development, industrialization, urbanization, and changes in individual and social values and norms are factors predisposing the fertility transition [10,11]. Industrial development is a driving force for fertility decline in developing countries where financial and technological help for family planning or birth control is provided by international organizations [12]. In addition, the declining birth rate is related to values and attitudes regarding marriage, lifestyle choices, parenthood, gender role attitudes, gender equity values, and so on [13,14]. Moreover, the recent labor market insecurity, higher unemployment of young adults, increased cost of living, nuclear family formations, gender equity issues, and inflexible gender roles in Korea are additional triggers for fertility pattern changes among Korean women [15,16].

To address the persistent low birth rate, the Korean government has formulated and implemented policies to create a more favorable environment for childbearing. They continue to provide support during pregnancy, medical assistance for childbirth to infertile couples, social protection for childbirth, childcare services, facilitating work-family reconciliation, childcare facilities at work, tax incentives, and support for child education [17]. In addition, international marriage migration has been encouraged by the government as a method of securing a labor force population and sociodemographic structure [8,18]. However, despite these concerns and policy interventions, it is reported that most of the government’s policies to boost fertility rate appear to be failing [19]. The government announced the Vision 2020 Plan to raise the fertility rate to 1.6 per woman (the average among OECD countries) by 2020 [20]; however, much work remains to be accomplished.

It is important to explain the phenomenon of Korea’s recent and continuing transition to the being the country with the lowest birth rate, and it is necessary to identify more relevant causes of low birth rate that may reflect in values held by women as well as the society. Since the number of Korean families who do not want to have a baby after marriage is growing, the initial strategy to overcome the low birth rate should be to encourage couples to have a first child. It is also essential to encourage subsequent childbirths in order to solve the demographic transition to an aged society in Korea. However, previous studies have attempted to identify the factors associated with low birth rate, rather than exploring distinct factors affecting first and subsequent childbirths [21,22]. In this study, we explored the following major research questions: “What are the factors that affect women’s first and subsequent births?” and “Are there differences between them?” The research hypothesis was that there would be differences in influencing factors between first and later childbirths. This study would contribute in proposing practical and veritable policies for overcoming the low birth rate in Korea by presenting differentiated strategies to encourage first and subsequent births.

## Aim

The objective of this study was to explore the factors influencing the low birth rate among married women in Korea using the National Survey data. Specifically, we explored and compared factors influencing women’s first childbirth and their subsequent childbirths.

## Methods

### Study design

This study was a secondary analysis of the “National Survey on Fertility and Family Health and Welfare”, which was a nationally representative survey conducted by the Korea Institute for Health and Social Affairs (KIHASA) [23].

### Setting and samples

The KIHASA has been conducting surveys since 1964 using a nationwide cluster sampling technique from the National Population and Housing Census. The survey was conducted in 2012 with 14,970 married women of childbearing age (aged 15–49 years) in Korea [24], and recently the data set was released for public researchers in 2015. In this study, we used the survey data of married women between the age of 19 and 39 years, who are generally considered to be actively fertile, to explore factors related to low birth rate. We included a total of 3,482 women in this study. The data is available as S1 Dataset.

### Ethical approval

This secondary analysis of the National Survey data was approved by the Institutional Review Board of Ajou university prior to initiating the study (IRB No. 16–233). All procedures performed in the study involving human participants were in accordance with the ethical standards of the institutional research committee and with the 1964 Helsinki declaration and its later amendments or comparable ethical standards.

### Measurements

In the survey, it was reported that the participants completed a self-report questionnaire including standardized items developed by KIHASA [24], which focused on marital and fertility status and behaviors of individuals and families belonging to different demographic and socioeconomic groups.

In this study, birth rate, measured by the number of childbirths, was chosen as a dependent variable. Independent variables included demographic characteristics (current age, age during the first childbirth, residential area, education level, religious orientation, employment status, type of occupation, and monthly household income) and psychosocial characteristics (perceptions about the value of marriage and children, social media influence, influence by current government policies, and expectations regarding future government policies).

### Data analyses

Data were analyzed using SPSS version 20.0 (SPSS Inc., Chicago, IL, USA). We used descriptive statistics to explain participants’ demographic and psychosocial characteristics. T-test and one-way ANOVA were used to analyze the differences in birth rate according to participant characteristics. In order to explore factors influencing women’s first childbirth and their subsequent childbirths, binary and ordinal logistic regression models were used with sampling region stratified bootstrap to correct probable regional sampling differences [25].

## Results

### Differences in birth rates according to women’s characteristics

The differences in birth rate in accordance with participants’ general characteristics are presented in Table 1.

**Table 1:** Differences in birth rate according to women’s characteristics (N = 3482).

Characteristics		n (%)	Birth rate		
			Mean ± SD	t or F	*p* (Scheffe)
***Demographic characteristics***					
Current age (33.77 ± 3.88)	20–29 years	534 (15.3)	0.79 ± 0.74	-23.51	< .001
	30–39 years	2947 (84.7)	1.66 ± 0.80		
Age during the first childbirth (27.16 ± 3.47)	10–19 years^a^	35 (1.2)	1.91 ± 0.70	133.56	< .001 (c < a, b)
	20–29 years^b^	2227 (74.8)	1.87 ± 0.63		
	30–39 years^c^	714 (24.0)	1.44 ± 0.54		
Residential area	Urban	2052 (58.9)	1.47 ± 0.84	-5.09	< .001
	Rural	1429 (41.1)	1.62 ± 0.87		
Education level	≤ High school	1267 (36.4)	1.69 ± 0.85	8.43	< .001
	College/university ≤	2213 (63.6)	1.44 ± 0.84		
Religious orientation	Yes	1473 (42.3)	1.58 ± 0.87	3.32	.001
	No	2008 (57.7)	1.49 ± 0.84		
Employment status	Full-time	1189 (34.1)	1.50 ± 0.82	1.06	.347
	Part-time	389 (11.2)	1.56 ± 0.86		
	No	1903 (54.7)	1.54 ± 0.87		
Type of occupation	Entrepreneur or professional^a^	542 (34.1)	1.41 ± 0.84	8.54	< .001 (a, b < d; b < c)
	Office worker^b^	522 (32.8)	1.36 ± 0.90		
	Service or sales worker^c^	380 (23.9)	1.57 ± 0.86		
	Manual labor worker^d^	147 (9.2)	1.70 ± 0.93		
Monthly household income ($4,237.90 ± 2,611.22)	< $3,000^a^	868 (25.0)	1.43 ± 0.89	10.94	< .001 (a < b, c)
	$3,001–4,999^b^	1608 (46.3)	1.53 ± 0.85		
	$5,000 ≤^c^	998 (28.7)	1.61 ± 0.81		
***Psychosocial characteristics***					
Perception about the value of marriage	No need to marry^a^	572 (16.5)	1.40 ± 0.92	11.01	< .001 (a, b < c, d)
	No matter what^b^	1570 (45.3)	1.50 ± 0.87		
	Better to marry^c^	1207 (34.7)	1.61 ± 0.80		
	Must marry^d^	120 (3.4)	1.71 ± 0.73		
Perception about the value of children	No need to have children^a^	546 (15.7)	1.44 ± 0.87	10.11	< .001 (a, b < c)
	Neutral belief about having children^b^	1283 (37.0)	1.48 ± 0.85		
	Must have children^c^	1641 (47.3)	1.60 ± 0.84		
Social media influence	Yes	690 (20.0)	1.40 ± 0.88	-4.49	< .001
	No	2766 (80.0)	1.56 ± 0.84		
Influence by current government policies	Support for childcare	244 (7.0)	1.53 ± 0.98	-0.60	.552
	Support and incentives for maternity	217 (6.3)	1.60 ± 0.95		
	No	3007 (86.7)	1.53 ± 0.84		
Expectation regarding future government policies	Support for childcare	571 (63.2)	0.87 ± 0.75	1.14	.320
	Expanded childcare facilities	256 (28.3)	0.82 ± 0.76		
	Support and incentives for maternity	77 (8.5)	0.74 ± 0.66		

Demographic characteristics revealed that birth rate was significantly higher among women in their 30s than those in their 20s (t = -23.511, *p* < .001), among those with their first childbirth occurring in 10s and 20s than in their 30s (F = 133.556, *p* < .001), among those living in rural areas than in urban areas (t = -5.092, *p* < .001), among those who graduated from high school than college/university (t = 8.427, *p* < .001), among those having a religious orientation (t = 3.318, *p* = .001), among those in occupations such as service, sales or manual labor workers than among entrepreneurs, professionals, or office workers (F = 8.544, *p* < .001), and among those with a relatively high household income (F = 10.936, *p* < .001). The birth rates did not significantly differ across women’s employment statuses (F = 1.058, *p* = .347).

As for psychosocial characteristics, women holding beliefs such as ‘people have to or better to marry’ had significantly higher birth rates than women who believed that ‘there is no need to marry or no matter what’ (F = 11.006, *p* < .001). Additionally, women who believed that ‘it is necessary to have children’ had significantly higher birth rates than women who believed that ‘it is not necessary to have children’ or those who held a neutral belief about having children (F = 10.110, *p* < .001). Women who stated that social media influenced their decisions about childbirth presented significantly lower birth rates (t = -4.493, *p* < .001). There were no significant differences in birth rates according to beliefs about current government policies (t = -0.596, *p* = .552) or expectations regarding future government policies (F = 1.139, *p* = .320).

### Factors influencing women’s first childbirth and subsequent childbirths

**Fig 1:**
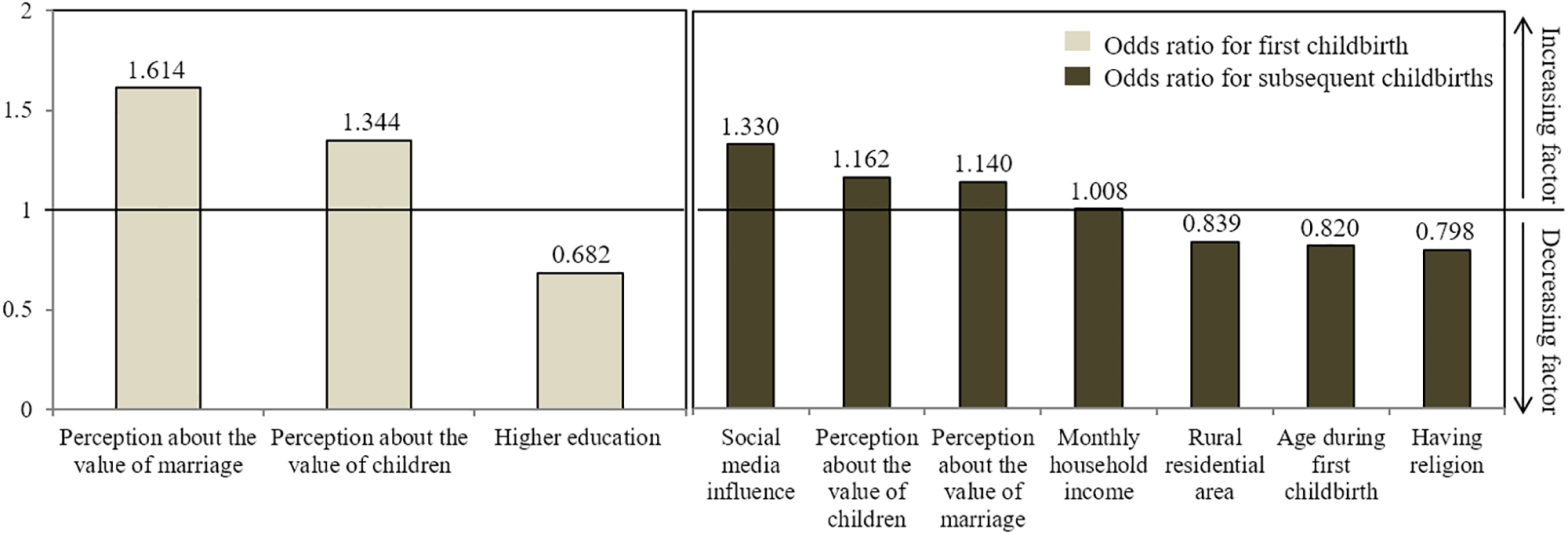
Comparison of influencing factors and odds ratios for women’s first and subsequent childbirths.

The factors that significantly influenced the first and the subsequent childbirths among married women are shown in Tables 2 and 3 and Fig 1. In order to explore the factors influencing childbirth, independent variables in the analysis were chosen if they were significant in the univariate analyses (i.e., residential area, education level, religious orientation, type of occupation, monthly household income, perceptions about the value of marriage and children, and social media influence) and selected if they were considered meaningful by the researchers (i.e., employment and influence by current government policies). Regarding age, participants’ age during the first childbirth was chosen as one of the independent variables since it was considered to be more relevant to birth rate than their current age was.

**Table 2:** Factors influencing women’s first childbirth (N = 1459).

Factor		Coefficient	S.E.	OR (95% CI)	*p*
Constant		-0.793	0.330		.022
Residential area (ref: urban residential area)	Rural	0.120	0.120	1.128 (0.891–1.428)	.337
Education level (ref: ≤ high school)	College/university≤	-0.383	0.135	0.682 (0.523–0.888)	.008
Religious orientation (ref: no religion)	Yes	0.028	0.118	1.028 (0.815–1.297)	.800
Employment status (ref: no employment)	Part-time	-0.151	0.191	0.860 (0.592–1.250)	.450
	Full-time	0.001	0.126	1.001 (0.787–1.282)	.992
Monthly household income (per $100)		0.004	0.003	1.004 (0.998–1.010)	.184
Perception about the value of marriage (score 1–4)		0.479	0.080	1.614 (1.379–1.889)	.001
Perception about the value of children (score 1–3)		0.296	0.082	1.344 (1.145–1.580)	.001
Social media influence (ref: no influence of social media)	Yes	-0.230	0.138	0.795 (0.606–1.042)	.105
Influence by current government policies (ref: no influence by government policies)	Incentives for maternity	0.425	0.252	1.529 (0.934–2.505)	.096
	Support for childcare	-0.093	0.207	0.911 (0.608–1.366)	.659

*Note* CI = confidence interval; OR = odds ratio

**Table 3:** Factors influencing women’s subsequent childbirths (ordinal logistic regression model; N = 2925).

Factor		Coefficient	S.E.	OR (95% CI)	*p*
Age during the first childbirth		-0.199	0.013	0.820 (0.799–0.840)	< .001
Residential area (ref: urban residential area)	Rural	-0.176	0.074	0.839 (0.723–0.964)	.020
Education level (ref: ≤ high school)	College/university≤	0.139	0.088	1.149 (0.969–1.387)	.094
Religious orientation (ref: no religion)	Yes	-0.226	0.076	0.798 (0.672–0.925)	.003
Employment status (ref: no employment)	Part-time	-0.092	0.127	0.912 (0.715–1.183)	.451
	Full-time	0.055	0.079	1.057 (0.908–1.232)	.495
Monthly household income (per $100)		0.008	0.003	1.008 (1.002–1.013)	< .001
Perception about the value of marriage (score 1–4)		0.131	0.055	1.140 (1.024–1.266)	.020
Perception about the value of children (score 1–3)		0.150	0.055	1.162 (1.050–1.305)	.006
Social media influence (ref: no influence of social media)	Yes	0.285	0.108	1.330 (1.081–1.631)	.004
Influence by current government policies (ref: no influence by government policies)	Incentives for maternity	-0.294	0.188	0.745 (0.517–1.064)	.056
	Support for childcare	-0.098	0.177	0.907 (0.625–1.276)	.529

*Note* CI = confidence interval; OR = odds ratio

Binary logistic regression was performed to identify the factors influencing women’s first childbirth (Table 2). The results showed that the odds ratios (ORs) increased for women having more positive perception about the value of marriage (OR = 1.614, 95% CI = 1.379–1.889) and for women having more positive perception about the value of children (OR = 1.344, 95% CI = 1.145–1.580). However, the OR decreased for participants who had graduated from college/university (OR = 0.682, 95% CI = 0.523–0.888). The influences of other variables on participants’ first childbirth were not significant.

Next, ordinal logistic regression was conducted to reveal significant influences on women’s subsequent childbirths (Table 3). It showed statistical significance (X^2^ = 392.623, *p* <.001), where the Cox & Snell R^2^ and Nagelkerke R^2^ were, respectively, 0.126 and 0.148, larger than 10%. Diverse variables demonstrated significant impacts on women’s number of childbirths, including women’s age during the first childbirth, residential area, religion, monthly household income, perceptions about the value of marriage and children, and social media. Women’s subsequent childbirths were positively associated with monthly household income (OR = 1.008, 95% CI = 1.002–1.013), perception about the value of marriage (OR = 1.140, 95% CI = 1.024–1.266) and children (OR = 1.162, 95% CI = 1.050–1.305) and social media (OR = 1.330, 95% CI = 1.081–1.631), but inversely associated with women’s age during first childbirth (OR = 0.820, 95% CI = 0.799–0.840), living in a rural area (OR = 0.839, 95% CI = 0.723–0.964) and religion (OR = 0.798, 95% CI = 0.672–0.925). Other variables of education level, employment status, and government policies showed no significant influence on women’s number of childbirths.

## Discussion

We explored the factors influencing childbirth of married women using recent National Survey data in Korea. Especially, we presented significant influences on women’s first childbirth and on their subsequent childbirths separately. It was revealed that the perceptions about the value of marriage and children positively influenced women’s first childbirth; further, having relatively higher education level had a negative impact on women’s first childbirth. Furthermore, along with the perceptions about the value of marriage and children, relatively higher monthly household income and social media had positive impacts on women’s subsequent childbirths. However, older age during the first childbirth, living in a rural area, and being religious had negative impacts on women’s subsequent childbirths.

In the present study, women’s perceptions about the value of marriage and children positively affected both the first and subsequent childbirths. In modern society, a woman’s marriage or parenthood has become an issue of personal preference [26]. Having positive perceptions about marriage and children would be the most basic predisposing factor in decisions about the family composition and childbirth. Meanwhile, higher education levels of women negatively affected the first childbirth in this study. The higher the education level of participants, the lower the intention of having the first child. This result was consistent with previous studies that the increase in the number of women who pursue higher education is closely related to the declining fertility rate [27], and the central reasons underlying the delay in having the first child are increases in women’s education, labor market participation, and changes in societal values [28,29]. Although higher education levels of women have undoubtedly had positive effects on modern industrial society, it may be quite difficult for women to give up their careers and choose childbirth without social support or adequate compensation.

Therefore, the first and the basic approach to overcome low birth rate, especially for the first childbirth, should be to create a facilitative social environment that fosters a positive perception about the value of family composition including children. Ideational shifts in the norms and values regarding children and parenthood and the broader culture and favorable attitudes toward family structures of a society would greatly contribute to people’s inclination toward childbirth in the long term. In addition, a prior study proposed that men’s participation in housework or involvement in child rearing is strongly associated with fertility rate [30]; therefore, it is necessary to make social efforts toward changing men’s perception and values about parenting. Additionally, there should be better social support systems and clear policies to help women pursue their academic and career aspirations without being negatively affected by the childbirth. Women’s employment and child rearing can be balanced when the policies and interventions are aimed to reduce the work-family conflicts. These include guarantees for paid parental leave, gender equality in parental leave, quantity and quality of childcare services for very young children, reducing gaps in the utilization of benefits from diverse social classes, and so on [31].

In this study, along with the perceptions about marriage and children, diverse variables such as age during the first childbirth, residential area, religion, monthly household income, and social media were derived as influencing factors on women’s subsequent childbirth. In fact, giving birth to two or more children is crucial in order to overcome the lowest-low fertility rate and to surpass the zero-population growth or replacement level of fertility for the future generation. Therefore, it is critical to consider multifaceted factors influencing women’s decisions to have their second or subsequent child.

Regarding women’s age, there has been notable change in age at first childbirth. According to a public report, the mean ages of Asian women at first childbirth were 31 years in Korea, 30.5 years in Singapore, 30.3 years in Japan, 29.8 years in Hong Kong, and so on [32]. As for Korean women born before 1950, most had given birth to their first child before they turned 25 years old, and this phenomenon was followed by an increasing number of births. In the 1990s, the average maternal age for women with more than three children was about 30 years; however, now, the average age of a woman during her first childbirth is more than 30 years old in Korea [33]. This phenomenon may be related to delayed marriage as an unmistakable trend in today’s world. Previous studies also showed that delayed marriage and childbirth are significant factors associated with the low fertility rate [19,34]. It is difficult but necessary to explore the various aspects of late marriage and childbirth of women and to build comprehensive strategies that support women’s marriage, pregnancy, childbirth as well as their career, employment, and so on.

Unlike our results, regarding demographic characteristics of women’s residential area and religion, a previous study in Finland showed that birth rate was the highest in rural areas and the lowest in the capital city [35]. Another study in the U.S. reported that women who answered that religion was very important in their life showed higher fertility than those saying religion was not important [36]. Perhaps particular religion or residence are associated with personal and family attitudes, and these attitudinal differences play a role in fertility decisions. Further studies on women’s demographic characteristics such as religion and residential area and its influence of mediation on childbirth are needed in the future.

In the present study, the government policy did not significantly influence women’s childbirth decision itself. However, government policy focused on financial assistance for childbirth or child rearing could encourage childbirth, because the result showed that economic factors such as monthly household income significantly influenced women’s decisions to have two or more childbirths. In previous studies, it was established that government policies should benefit parents to influence fertility rate; these include practical economic support (not only the benefits granted around childbirth, but also in-cash benefits covering childhood), extended leave periods with pay after childbirth, women’s labor market protection, support for work-family balance, monetary compensation, free and better access to childcare and early education facilities, and various forms of support for working parents during their children’s early years [15,37]. However, it is arduous to say that there are concrete recommendable government policies to directly improve birthrate in Korea, even though there have been a variety of attempts by the government [19]. Therefore, it is necessary to rethink the existing policies and generate more creative policies considering the practical needs and values of women. Future qualitative studies exploring their actual needs and barriers to childbirth in women are strongly suggested to revise policies for overcoming the low birth rate.

Moreover, in this study, the influence of social media was found to be a significant positive factor affecting women’s decision to have subsequent children. Social media has an incredibly powerful impact on people’s thoughts and behaviors [38]. Although there is considerable controversy over the impact of social media on women’s childbirth, it is true that women utilize the Internet or TV programs to learn about what to expect during childbirth, likely being validated in their choice to have a child [38]. Therefore, it is obviously necessary to improve awareness and encourage positive insights and behaviors about childbirth, and to develop strategies and practical tools to effectively utilize the social media as a strong medium to inculcate favorable attitudes toward childbirth and increase the future birthrate in the society.

As this was a secondary analysis using cross-sectional survey data, there are limitations in providing causal explanations for relationships between women’s characteristics and birth rates. In addition, we only investigated the factors affecting married women’s birth rate. Since the decision to have a child is not solely made by the woman, it is necessary for future studies to consider various influencing factors such as variables affecting men or gender-related roles, the perception of the birth of the family, and socio-cultural characteristics. Nevertheless, this study is meaningful in exploring factors influencing women’s first and subsequent childbirths separately, using National Survey on Fertility and Family Health and Welfare in Korea.

## Conclusions

Improving women’s awareness and facilitating positive perceptions about marriage and children are critical to increase the birth rate in Korea. Furthermore, financial and political support for maternity and childcare concerns and constantly striving to foster favorable attitudes toward childbirth and parenting via social media are crucial in increasing the birth rate in the future.

We suggest that further studies should evaluate the effectiveness of current political strategies regarding low birth rate. Such studies need to explore in depth, women’s perspectives on their needs pertaining to childbirth and those barriers to fulfilling their needs that may emerge from current government policies. Further interventions should be designed including strategies that consider the balance between work and family and the compatibility between career development and child rearing of married women.

## Supporting information

S1 Dataset Factors related to low birth rate among married women. (SAV)
